# Ketamine attenuates the glutamatergic neurotransmission in the ventral posteromedial nucleus slices of rats

**DOI:** 10.1186/s12871-017-0404-5

**Published:** 2017-08-23

**Authors:** Bao Fu, Chengxi Liu, Yajun Zhang, Xiaoyun Fu, Lin Zhang, Tian Yu

**Affiliations:** 1 Department of Critical Care Medicine, Affiliated Hospital of Zunyi Medical College, Zunyi, Guizhou China; 2 Guizhou Key Laboratory of Anesthesia and Organ Protection, Zunyi Medical College, Dalian Road, Zunyi, Guizhou 201 China

**Keywords:** Ketamine; glutamate, Postsynaptic currents, Patch clamp, VPM

## Abstract

**Background:**

Ketamine is a frequently used intravenous anesthetic, which can reversibly induce loss of consciousness (LOC). Previous studies have demonstrated that thalamocortical system is critical for information transmission and integration in the brain. The ventral posteromedial nucleus (VPM) is a critical component of thalamocortical system. Glutamate is an important excitatory neurotransmitter in the brain and may be involved in ketamine-induced LOC.

**Methods:**

The study used whole-cell patch-clamp to observe the effect of ketamine (30 μM–1000 μM) on glutamatergic neurotransmission in VPM slices.

**Results:**

Ketamine significantly decreased the amplitude of glutamatergic spontaneous excitatory postsynaptic currents (sEPSCs), but only higher concentration of ketamine (300 μM and 1000 μM) suppressed the frequency of sEPSCs. Ketamine (100 μM–1000 μM) also decreased the amplitude of glutamatergic miniature excitatory postsynaptic currents (mEPSCs), without altering the frequency.

**Conclusions:**

In VPM neurons, ketamine attenuates the glutamatergic neurotransmission mainly through postsynaptic mechanism and action potential may be involved in the process.

**Electronic supplementary material:**

The online version of this article (doi:10.1186/s12871-017-0404-5) contains supplementary material, which is available to authorized users.

## Background

Previous studies have demonstrated that thalamocortical system is critical for information transmission and integration in the brain [1, 2]. Our previous studies suggest that thalamocortical system is also involved in general anesthetics-induced loss of consciousness (LOC) [3–6]. The changes of glutamate transmission may play a critical role in general anesthetics-induced LOC. Rath et al. found that etomidate increases glutamatergic neurotransmission by inhibiting glutamate uptake in glial cells cultivated from the cortex of rats [7]. Our previous study found that etomidate decreases glutamatergic neurotransmission in thalamocortical neuronal network of rats [8].

Anaesthesia is used during surgery and other interventions to control pain, anxiety and consciousness [9]. Ketamine is a widely used intravenous anesthetic clinical settings and in emergencies, which can induce loss of consciousness (LOC). In addition, ketamine is often used in laboratory to anaesthetize animals. The glutamatergic receptor is an important target by which ketamine may cause LOC and analgesia in surgery [10–12]. Our previous study showed that ketamine inhibits the excitatory synaptic transmission of the neurons in the primary somatosensory cortex [13]. However, the effect of ketamine on glutamatergic neurotransmission in the ventral posteromedial nucleus (VPM) is still unknown. The present study used whole-cell patch-clamp to observe the effect of ketamine on glutamatergic neurotransmission in VPM.

## Methods

All experimental and surgical procedures were approved by Committees on Investigations Involving Animals in Zunyi Medical College, China. The experimental protocols were in accordance with the “Guide for the care and use of laboratory animals” in China (No. 14924, 2001) and current international standards.

### Materials

Ketamine was purchased from Gutian Pharma. Corp. (Fujian, China). 6,7-dinitro-quinoxaline-2,3(1H,4H)-dion (DNQX, a AMPA receptor antagonist), 2-Amino-5-phosphonovalerate-pharmacology(APV, a NMDA receptor antagonist),bicuculline (BIC,a blockade of GABA_A_ receptors), tetrodotoxin (TTX, a blockade of Na + channels) and strychnine (Str, a glycine receptors antagonist) were purchased from Sigma-Aldrich. Recording pipettes (tip diameter < 1 μM) were made from borosilicate glass capillaries (Sutter Instruments, Novato, CA) using the P-97 micropipette puller (Sutter Instruments, Novato, CA), as previous described [14].

### Slices

**Fig. 1:**
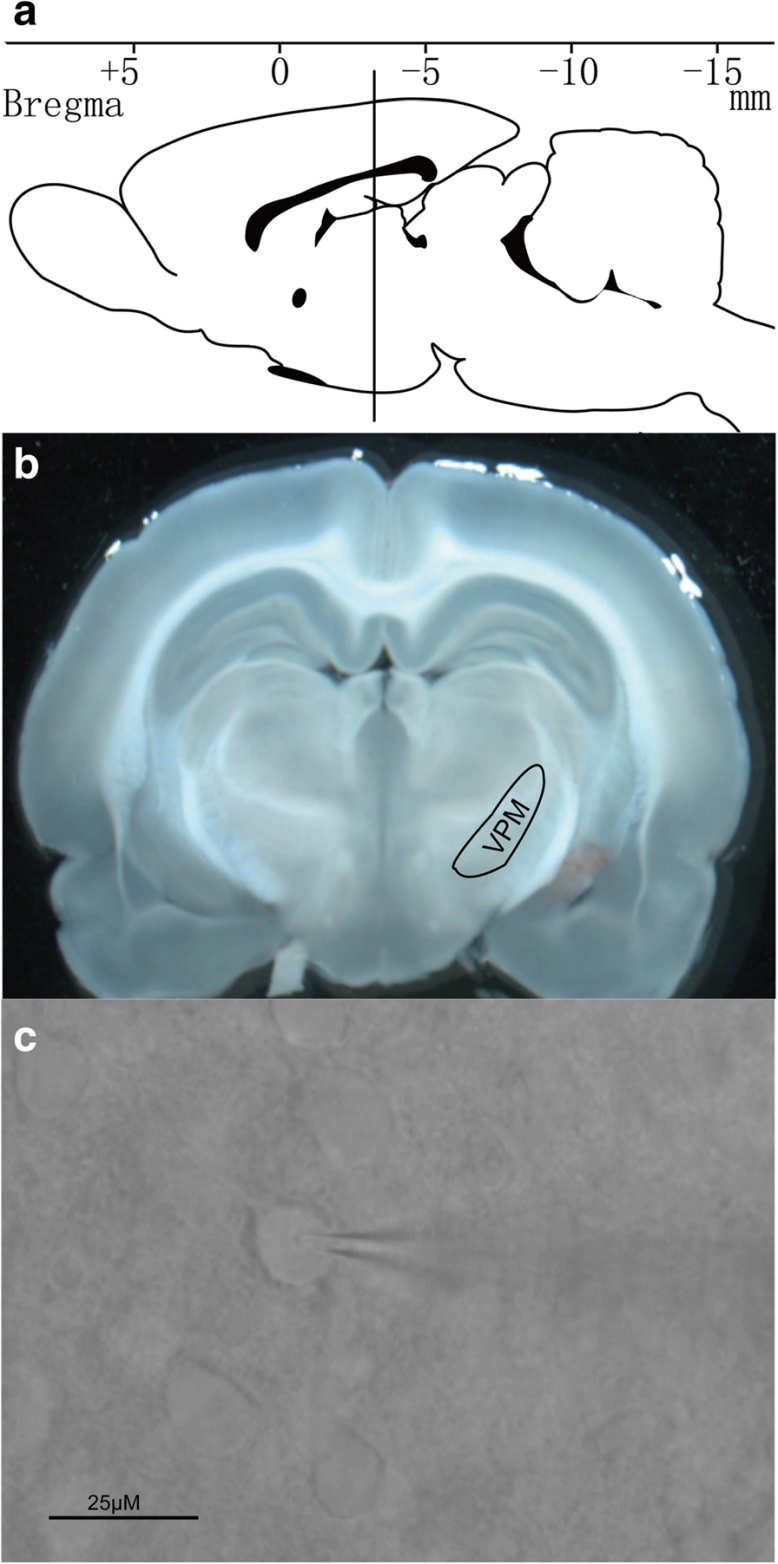
The position of VPM in the brain (**a**). A brain slice containing VPM was prepared from rats (**b**). A typical neuron was recorded in VPM (**c**)

Thirty Sprague–Dawley rats were purchased from animal center of the third military medical university (Chongqing, China). Thalamocortical slices were prepared as described previously [8] with a slight modification. Rats (7–15 days old) were deeply anesthetized with isoflurane and decapitated. After decapitated,the whole brain mass was isolated. The brain was then submerged in an ice-cold (0 °C) artificial cerebrospinal fluid (ACSF) containing (mM) NaCl 126, KCl 2.5,CaC1_2_ 2,MgSO_4_·7H_2_O_2_, NaHCO_3_ 25,NaH_2_PO_4_·2H_2_O 1.5,Glucose·H_2_O 10, (pH = 7.34–7.45 when bubbled with 95% O_2_–5% CO_2_). Then a block tissue containing VPM was detached from the brain using a sharp blade. The tissue was again immersed in an ice-cold ACSF and further sectioned with the HM 650 V Vibroslicer (Thermos Instruments, US) to manufacture brain slice with 250 μM thickness slices (Fig. 1b). Before whole-cell recording, the slices were incubated at 32 °C in ACSF for 1 h. ACSF was saturated with a mixture of 95% O_2_ and 5% CO_2_.

### Whole-cell recording

In whole-cell recording, VPM neurons were located by a BX51WI microscope (Olympus, Japan) with an infrared camera. The intracellular solution for recording EPSCs contained (mM) 140CsCl, 2 Na_2_ATP, 10 EGTA, and 10 HEPES (pH 7.4). The membrane potential was held at −65 mV. sEPSCs were recorded in the presence of 20 μM bicuculline and 1 μM strychnine. While mEPSCs were recorded in the presence of 20 μM bicuculline,1 μM strychnine and 0.5 μM TTX. The sEPSCs and mEPSCs were fltered at 3 kHz, digitized at 20 kHz with an HEKA EPC10 amplifier and Patch Master Software (HEKA InstrμMents, Inc., Lambrecht, DE), as previous described [15].

### Drug administration

In the present study, all drugs were perfused by a BPS-4 perfusion system (ALA, USA), as previous described [8]. Ketamine was dissolved into ACSF and the concentrations of ketamine in were 30 μmol/L、100 μmol/L、300 μmol/L、1000 μmol/L respectively. We started to record the current after a 10 min duration of drug application and the recording time lasted for 5 min.

### Data analysis

All currents were detected and analyzed using Mini Analysis 6 (Synaptosoft, Inc., Decatur, GA). For events recording, noise analysis was conducted for each cell and basal noise values during voltage-clamp recordings were <10 pA. The template wasselected visually according to the rise and decay phases of events, and the amplitude threshold was set to15 pA. The amplitude and frequency of currents were normalized to their mean value during the control period (5 min). The effects of drugs were expressed as % change (mean ± SEM) from pre-drug baseline, due to the large variability observed from neuron to neuron. The cumulative probability plots of the incidence of various inter-event intervals and amplitude, recorded in control conditions and during drug applications to the same neuro, as previous studies reported [16, 17]. We firstly run a normality test and the results show that the changes are normally distributed. Therefore, we used repeated measures one-way ANOVAs with post-hoc Bonferroni’s correction to evaluate discrepancies among groups, as previous study reported [15]. *P* < 0.05 was considered statistically significant.

## Results

**Fig. 2:**
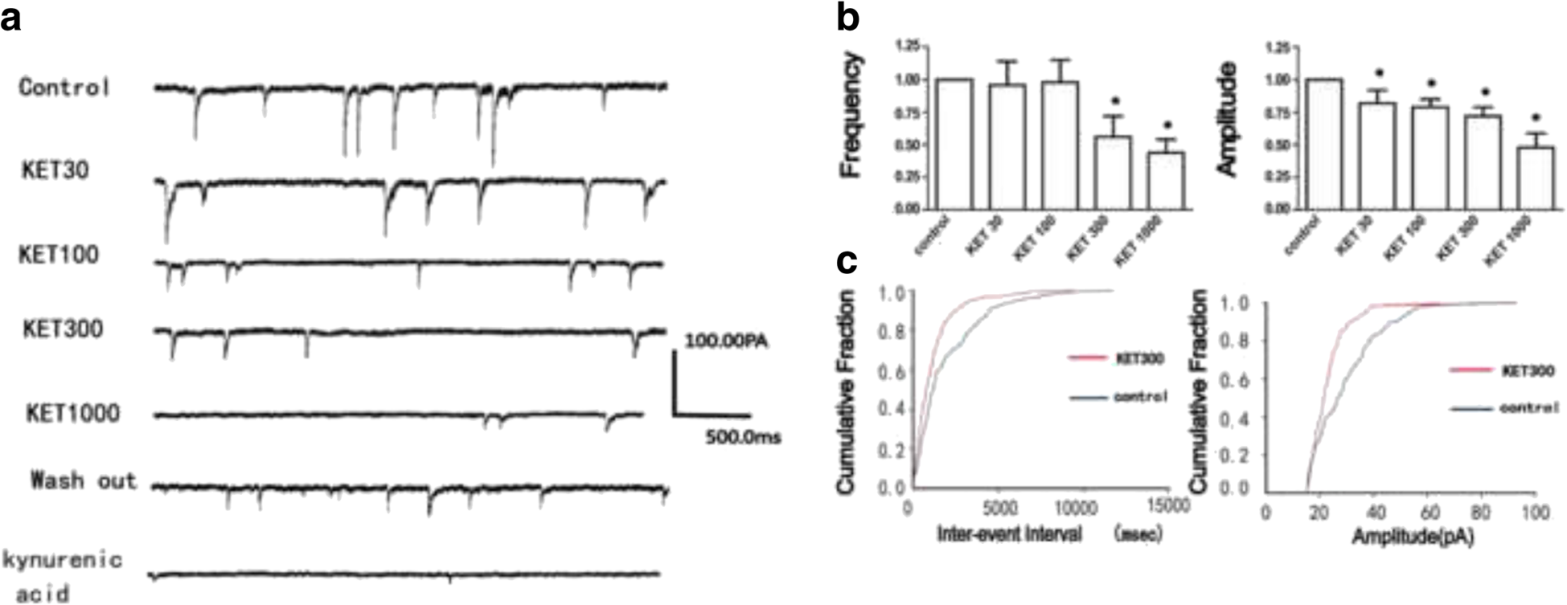
The original currents of sEPSCs recorded in a neuron in VPM (**a**). Ketamine (30 μM–1000 μM) decreased the amplitude, but only higher concentration of ketamine (300 μM and 1000 μM) suppressed the frequency of sEPSCs, ANOVA repeated measures, ^*^ *P* < 0.05, *n* = 8 (**b**). The cumulative probability plots of the incidence of various inter-event intervals and amplitude from the cell in A, as previous study reported [16, 17] (**c**)

In the present study, we recorded the target currents by antagonists and also verified the currents using antagonists, as shown in Fig. 2a.

### Effects of ketamine on sEPSCs of neurons in VPM nucleus

Figure 2a showed the typical currents of sEPSCs recorded in a VPM neuron. Ketamine (30 μM and 100 μM) did not significantly affect the frequency of sEPSCs (*P* > 0.05, *n* = 8), only higher concentration of ketamine (300 μM and 1000 μM) decreased the frequency of sEPSCs (Fig. 2b, *P*< 0.05, *n* = 8). However, ketamine (30 μM–1000 μM) decreased the amplitude, as shown in Fig. 2b (*P* < 0.05, *n* = 8). Figure 2c showed the cumulative probability plots of the incidence of various inter-event intervals and amplitude.

### Effects of ketamine on mEPSCs of neurons in VPM nucleus

**Fig. 3:**
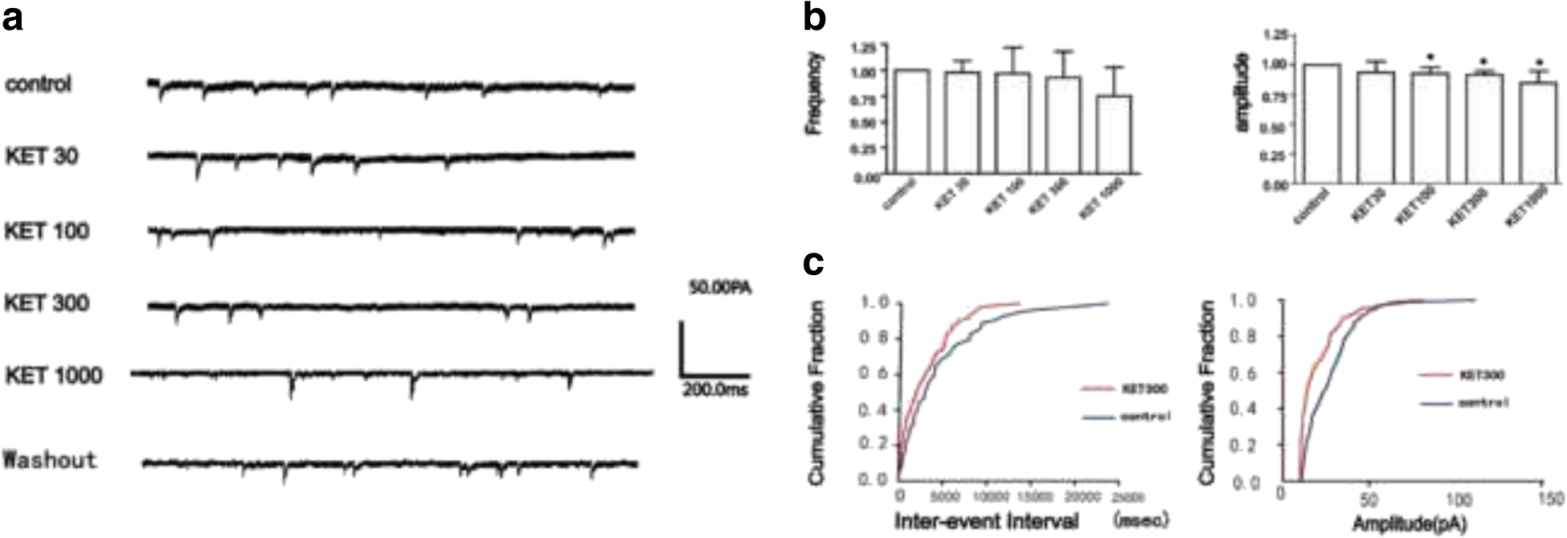
The original currents of mEPSCs recorded in a neuron in VPM (**a**). Ketamine decreased the amplitude, but did not alter the frequency of mEPSCs, ANOVA repeated measures, ^*^ *P* < 0.05, *n* = 8 (**b**). The cumulative probability plots of the incidence of various inter-event intervals and amplitude from the cell in A (**c**)

mEPSCs were recorded in the presence of TTX (0.5 μM) and bicuculline (BIC, 20 μM, Fig. 3a). Compared with control group, ketamine had no significant effect on the frequency (Fig. 3b, *P*>0.05, *n* = 8). However, ketamine (100 μM–1000 μM) decreased the amplitude of mEPSCs (Fig. 3b, *P*< 0.05, *n* = 8). The cumulative probability plots of the incidence of various inter-event intervals and amplitude were showed in Fig. 3c. After ketamine (1000 μM) perfusion, we started to perfuse with ACSF. The time of perfuse with ACSF was also 10 min and the recording time lasted for 5 min. However, we found the amplitude and frequency of sEPSCs did not recover to the baseline (control) after wash out, as shown in Additional file 1: Figure S1A and B. The amplitude of mEPSCs did not recover to the level of control, but the frequency recover to the recover to the level of control (Additional file 1: Figure S1A and B).

## Discussion

Previous studies have proved that inhibition of cortical pyramidal neurons and neurotransmitter transmission are likely to be involved in general anesthetics-induced LOC [18, 19]. Many researchers believe that the anesthetic-induced LOC is resulted from the interruption of the information integration between brain areas such as cortex, brainstem and thalamus [20–22]. NMDA and GABA receptors have been identified as two important pharmacological targets of anesthetics in both vivo and vitro studies [23]. Our previous study found that propofol inhibited neural activities in VPM and delayed the ascending signal transmission from VPM to primary somatosensory cortex [3]. A recent study showed that etomidate attenuates the glutamatergic neurotransmission in VPM [24]. However, etomidate increased glutamatergic neurotransmission by inhibiting glutamate uptake in In glial cells cultivated from the rat cortex [7]. It seems that the effects of anesthetics on neurotransmission in different brain areas may be different.

Ketamine is described as a dissociative anaesthetic and its use is limited because of its psychotic side-effects [25]. A recent study demonstrated that ketamine does not significantly affect the spatial and recognition memory or emotional reactivity of rats [9]. Ketamine can cause LOC both in human and rodents, the underlying mechanisms of ketamine on sensory information processing within the thalamocortical pathway are not fully understood. As we know, ketamine is a non-competitive inhibitor of NMDA receptor. Therefore, ketamine-induced LOC may be related with glutamatergic neurotransmission. The thalamocortical system has been proved as a vital area in sensory perception and modulating consciousness [2, 26]. Glutamatergic neurotransmission in thalamocortical system may play an important role in modulating sensory perception and consciousness.

Our results showed that ketamine (30 μM–1000 μM) decreased the amplitude of sEPSCs and higher concentration of ketamine (300 μM and 1000 μM) decreased the frequency of sEPSCs. Similarly, a previous study also found that ketamine (10 μM) depressed the peak amplitude of EPSC in cardiac vagal neurones in the nucleus ambiguous [27]. Ketamine did not alter the frequency of mEPSCs, but decreased the amplitude. The frequency of mEPSCs is considered as a measure of the presynaptic action and the amplitude is taken as a measure of the postsynaptic effect [28]. Thus, our results indicated that ketamine decreased the glutamatergic neurotransmission in VPM mainly through postsynaptic mechanism. However, ketamine decreased the frequency and amplitude of sEPSCs in the absence of TTX. TTX was used to block impulse propagation among VPM neurons. It seems that the impulse propagation is involved in ketamine-induced suppression on glutamatergic neurotransmission in VPM.

Presynaptic release of glutamate modulates excitatory neurotransmission through NMDA and AMPA receptors [29]. Consistent with us, ketamine (10 μM) decreased the amplitude of evoked EPSC in trigeminal sensory neurons [27]. Ketamine is a NMDAR activation dependent channel blocker. Ketamine probably attenuate glutamatergic neurotransmission through blocking the postsynaptic NMDA receptors in VPM neurons. Compared with the controls, the currents of sEPSCs and mEPSCs did not did not fully recover to baseline. This may be related to neurotoxicity of high concentration of ketamine [30].

## Conclusion

Ketamine attenuates glutamatergic neurotransmission mainly through postsynaptic action and the impulse propagation is involved in the process.

## Additional file

Additional file 1: Figure S1. The frequency (A) and amplitude of sEPSCs (B) did not recover to the level of control (*P* < 0.05, *n* = 8); the frequency (A) of mEPSCs recovered to the the level of control (C), but the amplitude did not recover to the baseline (D, *P* < 0.05, *n* = 8). (TIFF 1572 kb)

## Abbreviations

ACSF: artificial cerebrospinal fluid
BIC: bicuculline
LOC: loss of consciousness
mEPSCs: miniature excitatory postsynaptic currents
sEPSCs: spontaneous excitatory postsynaptic currents
TTX: tetrodotoxin
VPM: ventral posteromedial nucleus

## References

[1] Edelman GM (2003). Naturalizing consciousness: a theoretical framework. Proc Natl Acad Sci U S A.

[2] Tononi G, Koch C (2008). The neural correlates of consciousness: an update. Ann N Y Acad Sci.

[3] Zhang Y, Li Z, Dong H, Yu T (2014). Effects of general anesthesia with propofol on thalamocortical sensory processing in rats. J Pharmacol Sci.

[4] Zhang Y, He JC, Liu XK, Wang Y, Yu T (2014). Assessment of the effect of etomidate on voltage-gated sodium channels and action potentials in rat primary sensory cortex pyramidal neurons. Eur J Pharmacol.

[5] Li Z, Liu X, Zhang Y, Shi J, Xie P, Yu T (2014). Connection changes in somatosensory cortex induced by different doses of propofol. PLoS One.

[6] Zhang Y, Wang C, Zhang L, Yu T (2013). GABAA receptor in the thalamic specific relay system contributes to the propofol-induced somatosensory cortical suppression in rat. PLoS One.

[7] Rath M, Fohr KJ, Weigt HU, Gauss A, Engele J, Georgieff M, Koster S, Adolph O (2008). Etomidate reduces glutamate uptake in rat cultured glial cells: involvement of PKA. Br J Pharmacol.

[8] Fu B, Wang Y, Yang H, Yu T (2016). Effects of etomidate on GABAergic and glutamatergic transmission in rat Thalamocortical slices. Neurochem Res.

[9] Magalhaes A, Valentim A, Venancio C, Pereira M, Melo P, Summavielle T, Antunes L (2017). Ketamine alone or combined with midazolam or dexmedetomidine does not affect anxiety-like behaviours and memory in adult Wistar rats. Lab Anim.

[10] Chau PL (2010). New insights into the molecular mechanisms of general anaesthetics. Br J Pharmacol.

[11] Jin J, Gong K, Zou X, Wang R, Lin Q, Chen J (2013). The blockade of NMDA receptor ion channels by ketamine is enhanced in developing rat cortical neurons. Neurosci Lett.

[12] Franks NP (2008). General anaesthesia: from molecular targets to neuronal pathways of sleep and arousal. Nat Rev Neurosci.

[13] Yuan C, Zhang Y, Cao S, Wang Y, Fu B, Yu T (2016). Effects of ketamine on neuronal spontaneous excitatory postsynaptic currents and miniature excitatory postsynaptic currents in the somatosensory cortex of rats. Iranian journal of medical sciences.

[14] Chakraborty S, Elvezio V, Kaczocha M, Rebecchi M, Puopolo M (2017). Presynaptic inhibition of transient receptor potential vanilloid type 1 (TRPV1) receptors by noradrenaline in nociceptive neurons. J Physiol.

[15] Fu B, Yu T, Yuan J, Gong X, Zhang M (2017). Noradrenergic transmission in the central medial thalamic nucleus modulates the electroencephalographic activity and emergence from propofol anesthesia in rats. J Neurochem.

[16] Puopolo M, Bean BP, Raviola E (2005). Spontaneous activity of isolated dopaminergic periglomerular cells of the main olfactory bulb. J Neurophysiol.

[17] Haseneder R, Kurz J, Dodt HU, Kochs E, Zieglgansberger W, Scheller M, Rammes G, Hapfelmeier G: Isoflurane reduces glutamatergic transmission in neurons in the spinal cord superficial dorsal horn: evidence for a presynaptic site of an analgesic action. Anesthesia and analgesia 2004, 98(6):1718–1723, table of contents. 10.1213/01.ANE.0000112309.80017.3F 15155334

[18] Zhang Y, He JC, Liu XK, Zhang Y, Wang Y, Yu T (2014). Assessment of the effect of etomidate on voltage-gated sodium channels and action potentials in rat primary sensory cortex pyramidal neurons. Eur J Pharmacol.

[19] Bai D, Pennefather PS, MacDonald JF, Orser BA (1999). The general anesthetic propofol slows deactivation and desensitization of GABA(a) receptors. The Journal of neuroscience : the official journal of the Society for Neuroscience.

[20] Purdon PL, Pierce ET, Bonmassar G, Walsh J, Harrell PG, Kwo J, Deschler D, Barlow M, Merhar RC, Lamus C (2009). Simultaneous electroencephalography and functional magnetic resonance imaging of general anesthesia. Ann N Y Acad Sci.

[21] Velly LJ, Rey MF, Bruder NJ, Gouvitsos FA, Witjas T, Regis JM, Peragut JC, Gouin FM (2007). Differential dynamic of action on cortical and subcortical structures of anesthetic agents during induction of anesthesia. Anesthesiology.

[22] Ferrarelli F, Massimini M, Sarasso S, Casali A, Riedner BA, Angelini G, Tononi G, Pearce RA (2010). Breakdown in cortical effective connectivity during midazolam-induced loss of consciousness. Proc Natl Acad Sci U S A.

[23] Rudolph U, Antkowiak B (2004). Molecular and neuronal substrates for general anaesthetics. Nat Rev Neurosci.

[24] Fu B, Wang Y, Yang H, Yu T. Effects of etomidate on GABAergic and glutamatergic transmission in rat Thalamocortical slices. Neurochem Res. 2016; 10.1007/s11064-016-2042-6 27561291

[25] Morgan CJ, Mofeez A, Brandner B, Bromley L, Curran HV (2004). Acute effects of ketamine on memory systems and psychotic symptoms in healthy volunteers. Neuropsychopharmacology : official publication of the American College of Neuropsychopharmacology.

[26] Gili T, Saxena N, Diukova A, Murphy K, Hall JE, Wise RG (2013). The thalamus and brainstem act as key hubs in alterations of human brain network connectivity induced by mild propofol sedation. The Journal of neuroscience : the official journal of the Society for Neuroscience.

[27] Wang X, Gorini C, Sharp D, Bateman R, Mendelowitz D (2011). Anaesthetics differentially modulate the trigeminocardiac reflex excitatory synaptic pathway in the brainstem. J Physiol.

[28] Banks MI, Pearce RA (1999). Dual actions of volatile anesthetics on GABA(a) IPSCs: dissociation of blocking and prolonging effects. Anesthesiology.

[29] McBain CJ, Mayer ML (1994). N-methyl-D-aspartic acid receptor structure and function. Physiol Rev.

[30] Shibuta S, Morita T, Kosaka J, Kamibayashi T, Fujino Y (2015). Only extra-high dose of ketamine affects l-glutamate-induced intracellular ca(2+) elevation and neurotoxicity. Neurosci Res.

