# Supplementary figures and images for: Ketamine attenuates the glutamatergic neurotransmission in the ventral posteromedial nucleus slices of rats
Source: BMC Anesthesiol. 2017 Aug 23;17:111. doi: 10.1186/s12871-017-0404-5 (PMC5569565; doi:10.1186/s12871-017-0404-5)

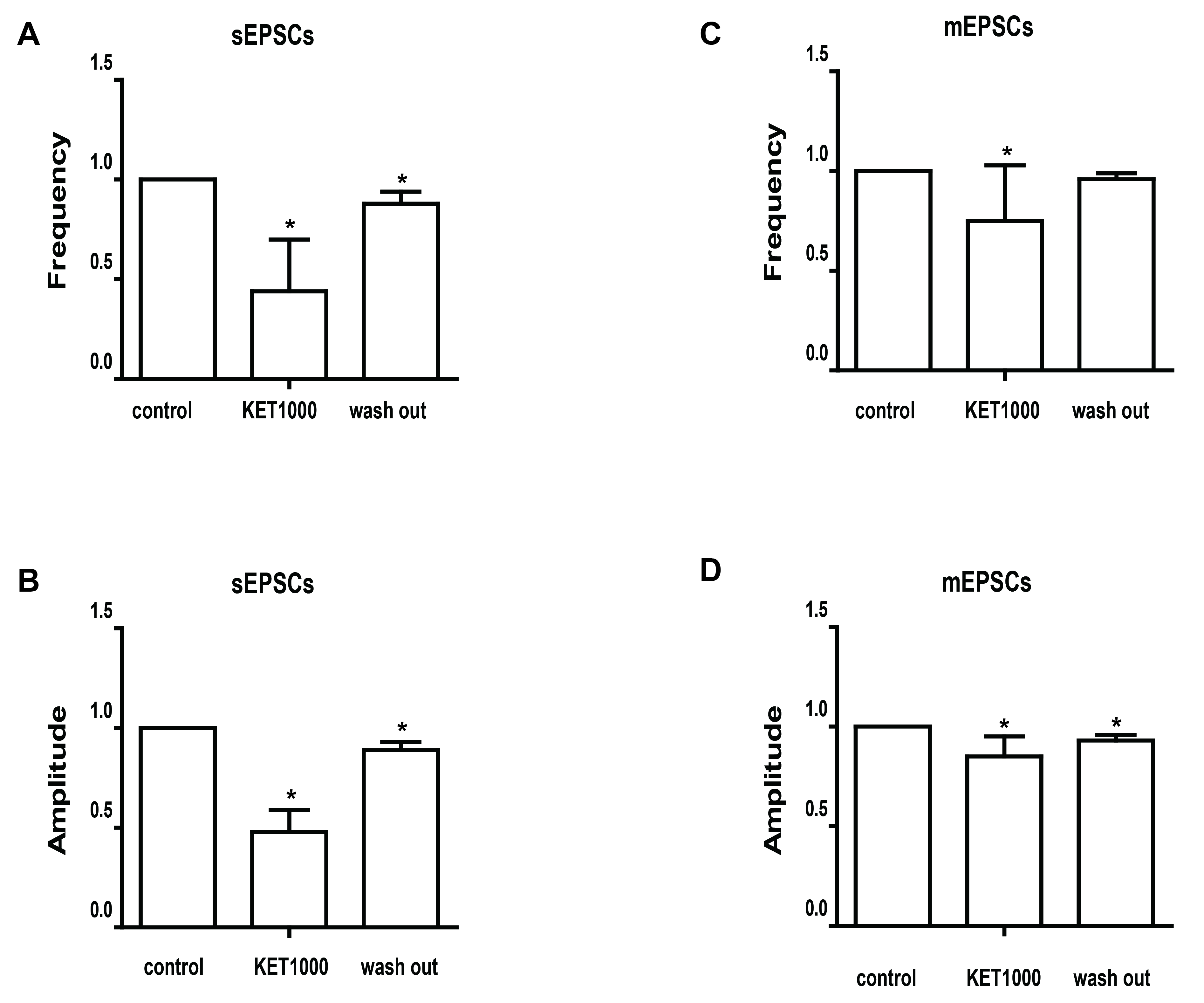

Supplement: Additional file 1: Figure S1. — The frequency (A) and amplitude of sEPSCs (B) did not recover to the level of control (P < 0.05, n = 8); the frequency (A) of mEPSCs recovered to the the level of control (C), but the amplitude did not recover to the baseline (D, P < 0.05, n = 8). (TIFF 1572 kb) [file 12871_2017_404_MOESM1_ESM.tif]
